# Vegetable Peel Waste for the Production of ZnO Nanoparticles and its Toxicological Efficiency, Antifungal, Hemolytic, and Antibacterial Activities

**DOI:** 10.1186/s11671-016-1750-9

**Published:** 2016-12-08

**Authors:** T. V. Surendra, Selvaraj Mohana Roopan, Naif Abdullah Al-Dhabi, Mariadhas Valan Arasu, Gargi Sarkar, K. Suthindhiran

**Affiliations:** 1 Chemistry of Heterocycles & Natural Product Research Laboratory, Department of Chemistry, School of Advanced Sciences, VIT University, Vellore, 632 014 Tamilnadu India; 2 Department of Botany and Microbiology, Addiriyah Chair for Environmental Studies, College of Science, King Saud University, P. O. Box 2455, Riyadh, 11451 Saudi Arabia; 3 Marine Biotechnology and Byproducts Laboratory, Department of Biomedical Sciences, School of Bioscience and Technology, VIT University, Vellore, 632 014 Tamilnadu India

**Keywords:** Zinc oxide (ZnO) nanoparticle (NP) synthesis, Photocatalytic activity, Antifungal activity, In vitro hemolytic activity, Antibacterial activity

## Abstract

Zinc oxide (ZnO) nanoparticles (NPs) are important materials when making different products like sun screens, textiles, and paints. In the current study, the photocatalytic effect of prepared ZnO NPs from *Moringa oleifera* (*M. oleifera*) was evaluated on degradation of crystal violet (CV) dye, which is largely released from textile industries and is harmful to the environment. Preliminarily, ZnO NP formation was confirmed using a double beam ultraviolet visible (UV-Vis) spectrophotometer; further, the NP size was estimated using XRD analysis and the functional group analysis was determined using Fourier transform infrared (FT-IR) spectroscopy. The morphology of the synthesized NPs was found to be a hexagonal shape using SEM and TEM analysis and elemental screening was analyzed using EDX. ZnO NPs were shown sized 40–45 nm and spherical in shape. The degradation percentage of ZnO NPs was calculated as 94% at 70 min and the rate of the reaction –k = 0.0282. The synthesized ZnO NPs were determined for effectiveness on biological activities such as antifungal, hemolytic, and antibacterial activity. ZnO NPs showed good antifungal activity against *Alternaria saloni* and *Sclerrotium rolfii* strains. Further, we have determined the hemolytic and antibacterial activity of ZnO NPs and we got successive results in antibacterial and hemolytic activities.

## Background

Synthetic dyes and other contaminated stuffs are the waste products which are being discharged from many industries. These effluents are mostly toxic to nature, which also results in various health effects. Nowadays, the decrease of the effect of these dyes on nature is stepping forward by using many degradation methods like physical methods, chemical coagulation, ion exchange, and other methods [1]. Normally, dyes are very stable chemical pollutants, so traditional treatment methods are unsuccessful for the degradation of dyes [2]. These textiles dyes have many reactive ingredients on the chemical oxidation process as well as stable photocatalytic agents. In recent years, the importance of the photocatalyst in the process of dye degradation has been explained by many researchers to overcome the drawback [3]. Dye removal from waste water treated using green synthesized nanoparticles (NPs) by different methodologies is gaining more interest. Considerably, nanotechnology is one of the emerging fields among the various studies with vast applicational properties [4]. The metal and metal oxide NPs have attractable properties like biological, electronic, magnetic, and photocatalytic activity [5]. Due to the size and morphological effect, the metal oxide NPs can be attached to the surface of toxic chemicals [6]. The direct band gap at room temperature of zinc oxide (ZnO) NPs is 3.3 eV and the excitation binding energy is 60 meV. Due to these encouraging specific characteristics, ZnO NPs were suggested as good photocatalytic agents [7, 8]. On the other hand, ZnO NPs have abundant biological properties such as antimicrobial [9, 10], antioxidant [11], anticancer, and other activities [12–15].

The synthesis of ZnO NPs through the biological method using enzymes, microorganisms, and plants and their extracts has been suggested as a cost-free method. This eco-friendly method for ZnO NP synthesis attracts more importance and is an alternative to the chemical and physical methods [16], due to the avoidance of the use of toxic chemicals and high energy ingredients in the synthesis process. Plant extracts actively participate in the bio-reduction process to convert the metal ions to metal and metal oxide NPs [17–19]. Basically, ZnO NPs are quality photocatalysts due to their capacity to generate the energy by reactive oxygen species (ROS) [14]. Green synthesized ZnO NPs play a major role against the degradation process of industrial dyes due to the photocatalytic effect [20]. In this regard, we have effectively synthesized the ZnO NPs from *M. oleifera* peel extract (MFPE). The photocatalytic degradation of high concentrations of crystal violet (CV) has been investigated for the first time in detail. Also, the antifungal, hemolytic, and antibacterial activities of the ZnO NPs were determined.

## Methods

### Materials

**Fig. 1:**
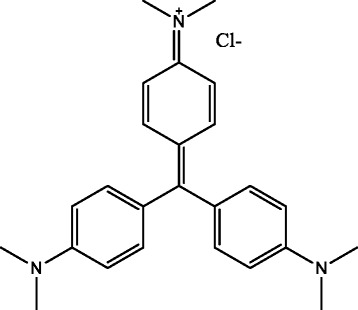
Structure of CV

*M. oleifera* was procured in and around Vellore local market (12.9202° N, 79.1333° E), Tamil Nadu, India. Further, it was identified and authenticated by Agricultural University, Coimbatore as BSI/SRC/S/23/2013-14/tech. 1116. Zinc acetate and C_25_N_3_H_30_Cl (CV) (Fig. 1) were procured from Sigma Aldrich, India. Clinical bacterial strains *S. aureus* (ATCC 4163) and *E. coli* (ATCC 25922) were used. Potato dextrose broth was procured from Hi-media Laboratories, Maharashtra, India and throughout the experiment Milli Q water was utilized without any further purification.

### Methanolic Extract Preparation of *M. Oleifera* Peel

Drum sticks were cleaned by using distilled water and the flesh removed. The separated peel was dried under room temperature and milled as a fine powder. The source was extracted by a technique of maceration with methanol as a solvent. It was further distilled and the extract was collected and stored in a refrigerator for future progress.

### Green Synthesis of ZnO NPs Using *M. Oleifera* Peel

In the microwave-assisted synthesis, 1 mmol of Zn(OAc)_2_ stock solution was prepared and methanolic extract dissolved in milli Q water. The MFPE (20 mL) solution was added to 80 mL of Zn(OAc)_2_ in the ratio of 20:80. After proper mixing, this mixture reaction placed in oven at the microwave power of 300 W for the 5 min irradiation time [21]. The preliminary conformation of ZnO NPs was identified by ultraviolet visible (UV-Vis) analysis in the range of 200–800 nm at different time intervals of irradiation time. The reaction mixture of ZnO NPs was subjected for centrifugation for 10 min at 10,000 rpm. Further pellets were collected and supernatants were discarded. This process was repeated for three teams in order to remove impurities using distilled water. Further, the obtained pellets were calcinated at 400 °C and 600 °C.

### Characterization/Instrumentation Used During Experiments

Synthesis of ZnO NPs and extraction of secondary metabolites were performed in UWave - 1000 multifunction microwave workstation. Schimazu UV-Vis Spectroscopy (UV-1800) was used to record UV - Vis spectrum. FTIR was recorded in Bruker Alpha T model whereas XRD has been analyzed with the help of Bruker D8 instrument. SEM/EDAX (JEOL JSM-6390LV) and TEM (Philips; CM 200) were used to find the morphological and size of the ZnO NPs. Horiba nanoparticle analyzer was utilized for Zeta potential result.

### Photocatalytic Degradation of CV

Green synthesized ZnO NPs allowed for testing the efficiency on CV dye degradation. The reaction mixture was made by interacting 1 mg/L of CV solution with 5 mg/L of ZnO NPs. The photocatalytic degradation was determined using UV-Vis analysis at a range of 254 nm based on the degradation of CV at 15 min time intervals [1]. The accurate degradation rate of reaction:

1 $$ ln\left(C/{C}_0\right) = - kt $$

Where final concentration = C, starting concentration = C_0_, rate constant = *k*, and t = time.

### ZnO NPs: Antifungal Studies

ZnO NP antifungal studies were done using the CLSI method and two phytopathogenic fungal strains *Alternaria saloni* and *Sclerrotium rolfii* were used for the determination of the activity. A total of 100 mL of potato dextrose broth was autoclaved and 1 mL fungal cultures were added to the broth. A 1 mg/mL concentration was added to test samples and placed in an incubator for 7–10 days at 37 °C followed by continuous stirring at 120 rpm. After incubation, the broth was filtered and the biomass was collected for drying [22]. The weight of the dried biomass was taken and analyzed for the study and carbendazim was used as a standard.

### ZnO NPs: In Vitro Hemolysis

We collected a healthy volunteer human sample (B^+^ blood male) and stored it in a sterile container. The erythrocyte suspension was obtained by the centrifugation of the blood sample at 1500 rpm for 5 min. The obtained suspension was washed with phosphate buffered saline (PBS) at pH 7.4 for pure erythrocyte suspension for hemolytic activity. The synthesized ZnO NPs were distributed in PBS under a sonication process and separated as different concentrations of 25, 50, 75, and 100 μL. The NP solution was added to the human erythrocytes diluted in PBS. The red blood cells (RBCs) mixed in PBS were used as a negative control and Triton- × 100 was used as a positive control. After 30 min incubation at room temperature, the samples were centrifuged at the same rpm as mentioned above for 10 min. The obtained supernatant was used for the determination of toxicity on RBCs at 540 nm [23]. The hemolysis percentage was calculated using (As – Anc/Apc – Anc) × 100, where As is denoted as sample absorbance, Apc is denoted as positive control absorbance, and Anc is denoted as negative control absorbance.

### ZnO NPs: Antibacterial Studies

We processed this study against the two standard clinical strains such as *S. aureus* (gram positive) and *E. coli* (gram negative) using a well diffusion method [24]. The nutrient agar plate was used for the inoculation of both bacterial strains. The bacterial strains were swabbed using the cotton buds or swab and about 7 mm diameter of well was made using a well borer. About 25 μL of synthesized ZnO NPs were added into the well and plates placed in an incubator at room temperature for 24 h. Here we used Amoxicillin -1 as the positive control and water as the negative control to perform the study.

## Results and Discussion

### UV-Vis Spectroscopy

**Fig. 2:**
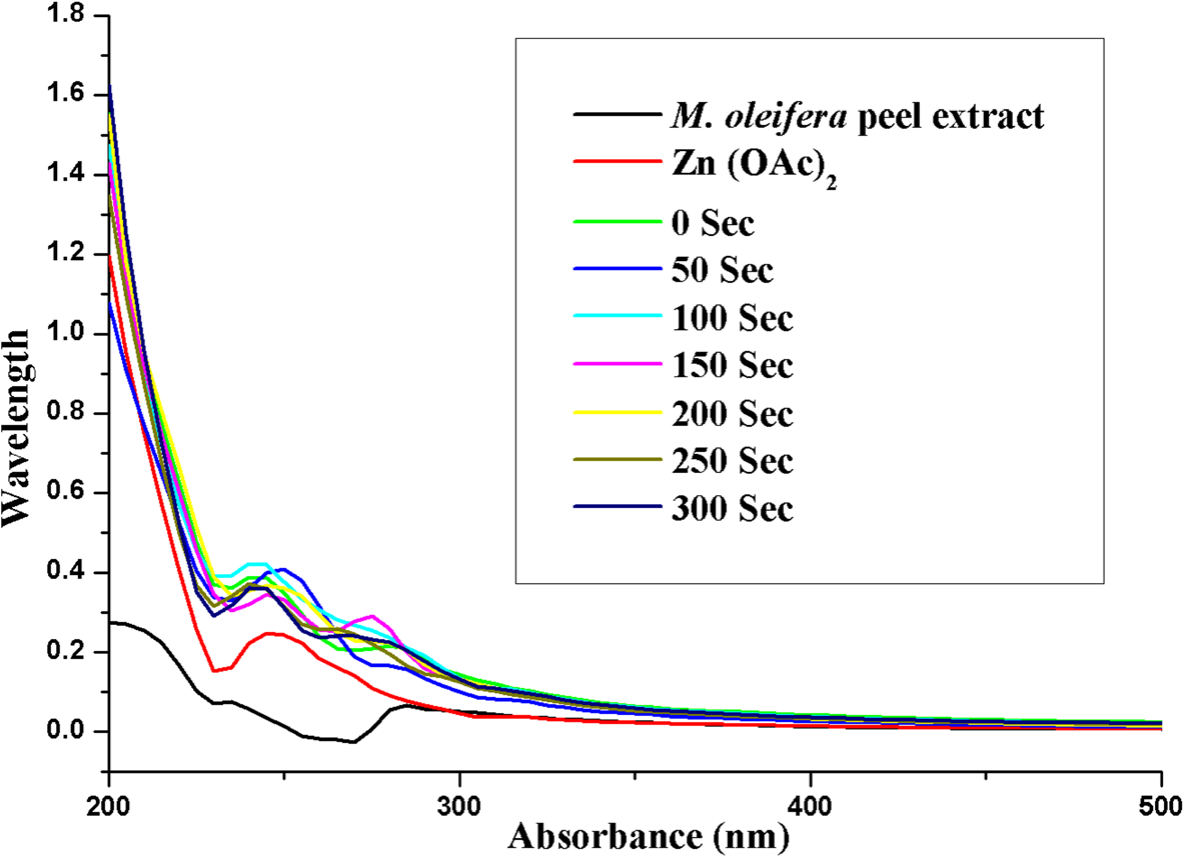
UV-Vis absorption spectra of Zno NP formation

The UV-Vis analysis was used for the identification of optical property of ZnO NPs. ZnO NP formation was confirmed with the absorption peak around 300 nm as shown in Fig. 2. The high exciton binding energy of ZnO NPs was responded at 270 nm as clear absorption band. As a results 150 s was observed as optimized time for the formation of ZnO NPs.

### ZnO NP XRD Studies

**Fig. 3:**
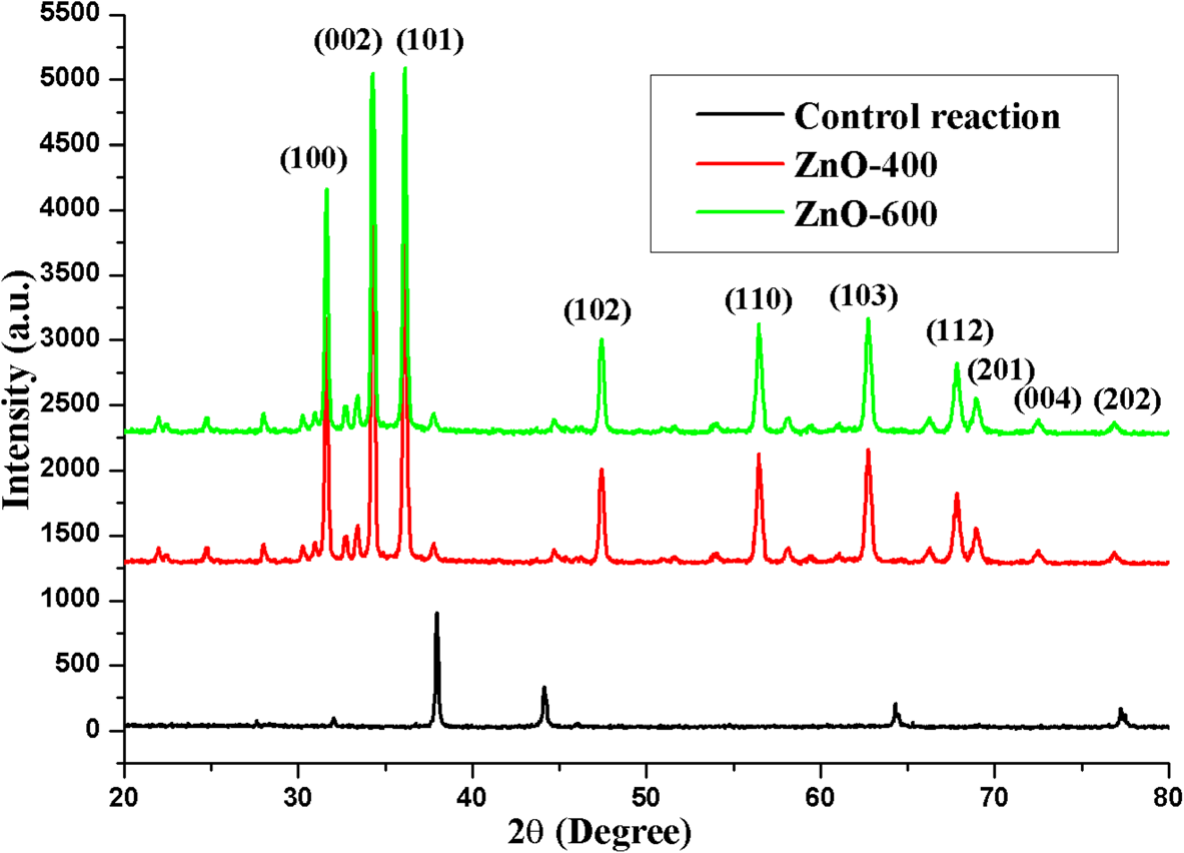
X-ray diffraction pattern of ZnO NP formation

The XRD results in several peaks at 31.73°, 34.43°, 36.21°, 47.55°, 56.56°, 62.82°, 66.30°, 67.92°, 69.02°, 72.50°, and 76.97° and these correspond to (100), (002), (101), (102), (110), (103), (200), (112), (201), (004), and (202) planes of ZnO NPs (Fig. 3). The XRD plane values were in agreement with JCPDS no. 89-7102.8. The anisotropic growth and crystallites orientation of the ZnO NPs were indicated by a high intensity peak at (101). The structure of the ZnO NPs was confirmed as a hexagonal wurtzite structure and the crystalline nature of the NPs was confirmed by stiff and narrow diffraction peaks. Scherrer’s formula was used for calculate crystalline size which resulted in 40–45 nm.

2 $$ \mathrm{D} = \mathrm{K}\uplambda /\upbeta\ COS\ \uptheta $$

Where the size of the particle is denoted as D, Scherer’s (0.94) constant is denoted as K, Bragg’s equation (2dsinθ = nλ), Wavelength is denoted as be λ, FWHM is denoted as β, and the diffraction angle is denoted as θ.

### ZnO NPs: FT-IR Analysis

**Fig. 4:**
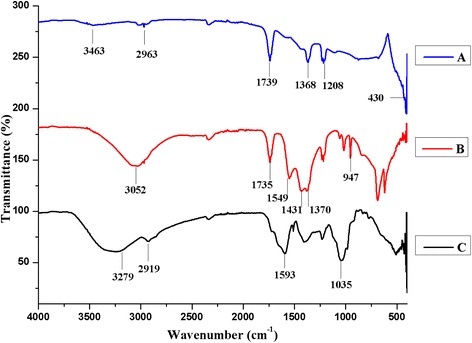
FT-IR spectrum: (**a**) ZnO NPs, (**b**) Zn (OAc)_2_, and (**c**) MFPE

FT-IR analysis was carried out using the KBr method for the detection of functional groups which are present in the synthesized ZnO NPs and MFPE (Fig. 4). The ZnO bond bending peak appeared in the range of 430 cm^−1^ and the metal oxygen peak was observed in the region 400–600 cm^−1^. The peaks appeared at 3462, 2963, 1739, and 1585 cm^−1^ and corresponded with phenol O-H, C-H stretching, C = O, and 1° amine, respectively. The intense bands observed at 1368 cm^−1^ indicate the rock C-H of the alkane group and 1208 cm^−1^ is a region of C-N stretching of aliphatic amines.

FT-IR spectra also confirm the functional groups of MFPE with the absorption band at 3279, 2919, 1593, and 1035 cm^−1^, respectively, as shown in Fig. 4. These peaks occurred due to amino acids, alkaloids, flavonoids, and phenolic acids. Further, the Zn(OAc)_2_ structure was conformed with the absorption peaks at 3000–3100, 1735, 1549, 1431, 1379, and 947 cm^−1^, respectively.

### SEM, TEM, EDAX, and Histogram Analysis

**Fig. 5:**
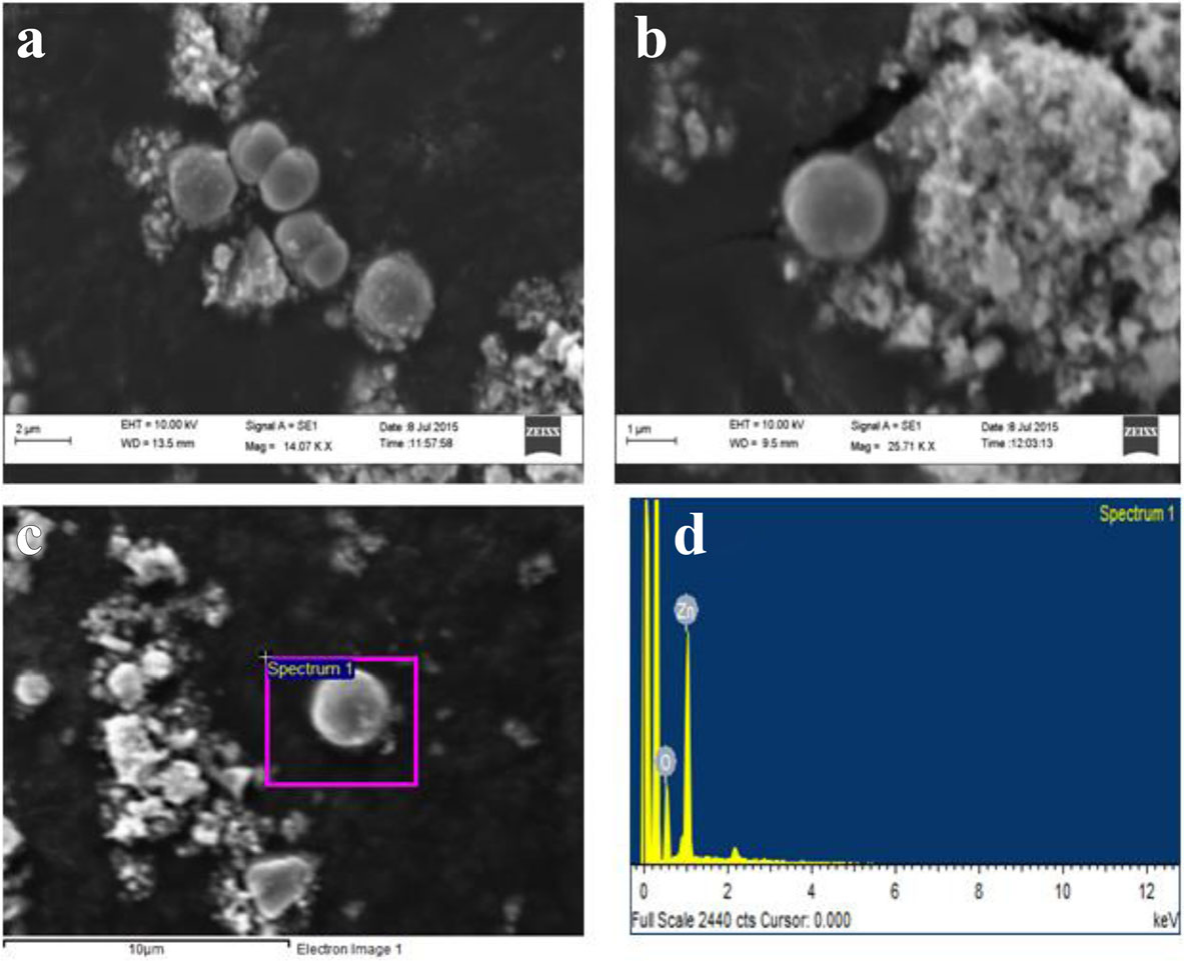
(**a**–**c**) SEM and (d) EDX spectrum of ZnO NPs

**Fig. 6:**
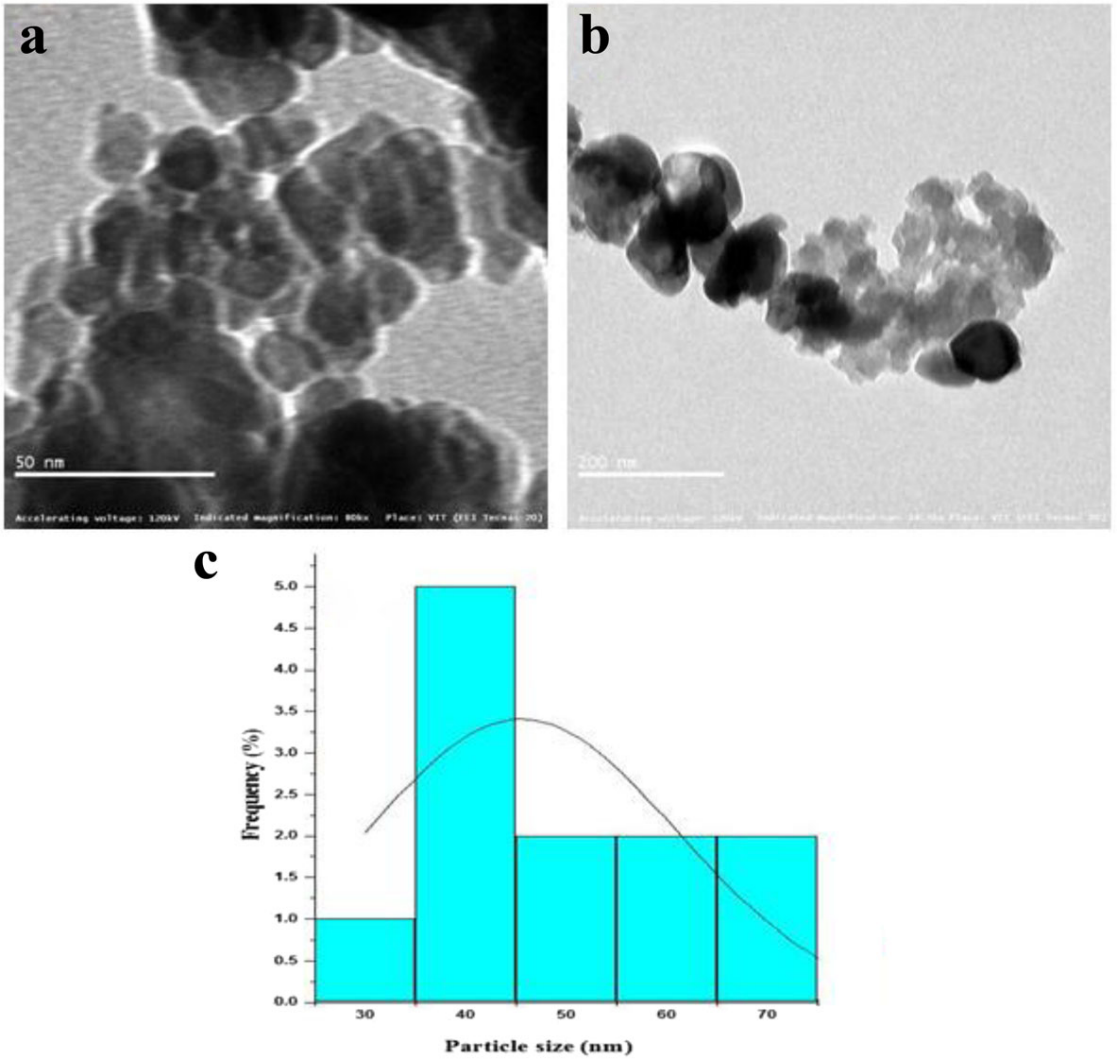
**a**, **b** TEM images of ZnO NPs. **c** Particle size histogram

We performed a microscopic analysis to identify the size and shape of the synthesized particles. The observed result stated that synthesized ZnO NPs were spherical in shape (Fig. 5a–c). The chemical profile of ZnO NPs was analyzed using EDAX which results in 72.15% of zinc and 27.85% of oxygen present and the atomic percentage of zinc at 61.20% and oxygen at 38.80% (Fig. 5b). Figure 6a and b show the agglomerated ZnO NPs average size to be 40–45 nm, as shown in Fig. 6c, and the highest distribution percentage of the ZnO NPs histogram is 40 nm.

### Photocatalytic Efficiency of ZnO NPs

The band gap will increase the ROS by the NPs when treating with the irradiation light of frequency within or above of their range. The wavelength and surface electrons of the nanoparticles will get energy and move from ground level to an excited level, as the result a new band will form [25]. The formation of valence bands (VBs) and conduction bands (CBs) occurs due to the mechanistic properties with the positive charged holes and negative charged electrons, respectively. The positive charged holes react with water; it leads to hydroxyl radical (.OH^–^) formation and acts as a strong oxidizing agent. The radical anions (.O^−2^) are formed due to the reaction between photogenerated negative charged electrons and molecular oxygen. These two species are commonly known as ROS. ROS were a very important species to the photocatalytic activity of the NPs [26].

**Fig. 7:**
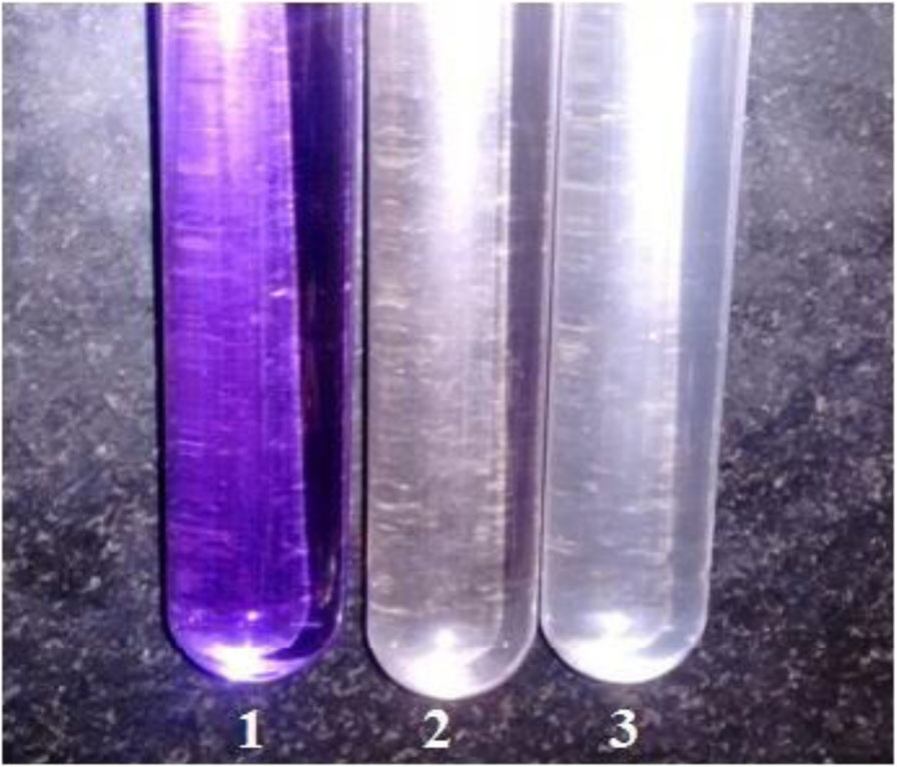
Color and effect degradation of CV dye by ZnO NPs: (1) Dye, (2) Dye + ZnO NPs at 0 min, and (3) Dye + ZnO NPs at 70 min

**Fig. 8:**
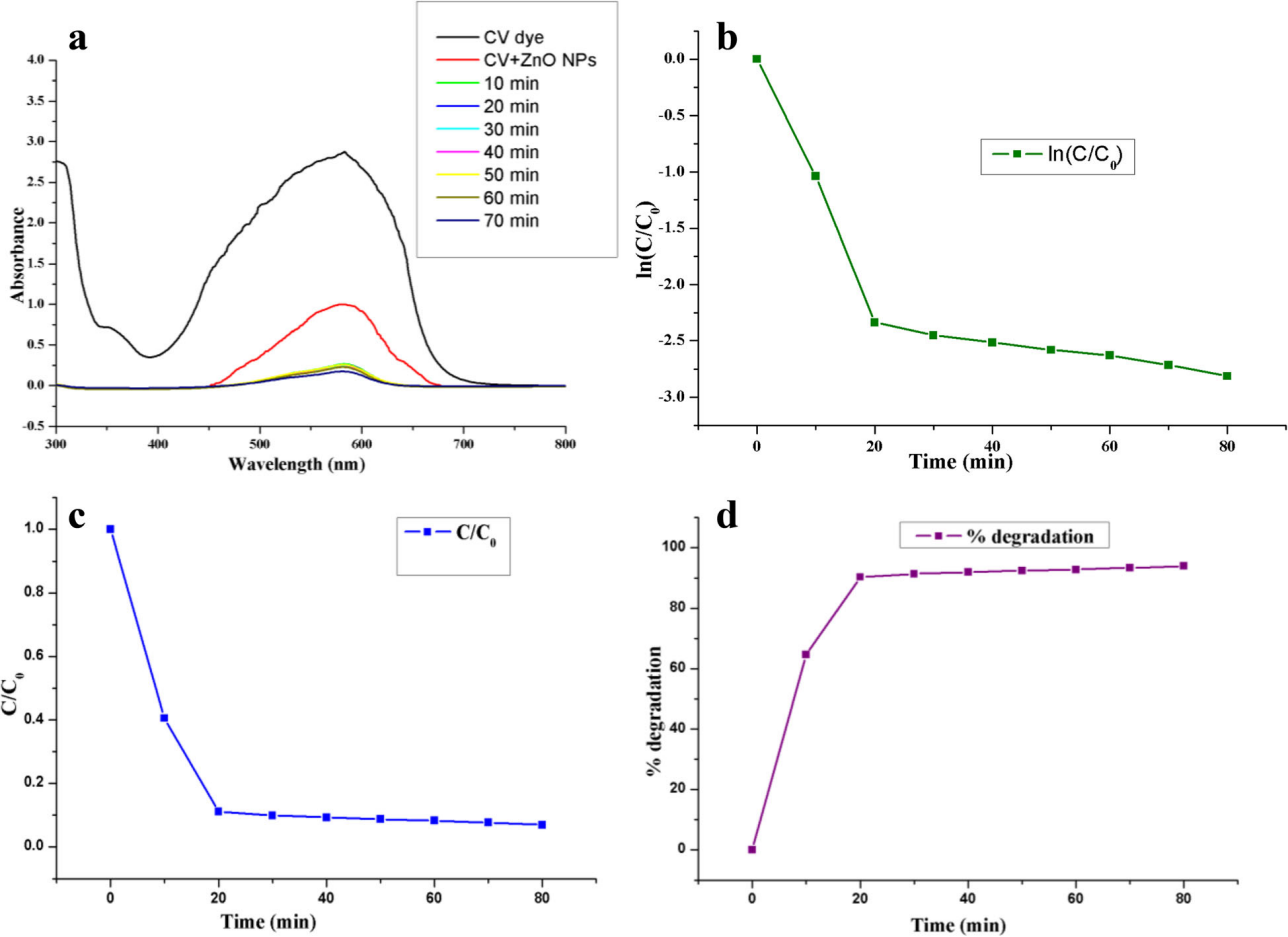
**a** Photocatalytic degradation of CV dye by ZnO NPs. **b** Rate constant (ln C/C_0_). **c** C/C_0_. **d** % degradation

We utilized a photoreactor with a multilamp at exactly 365 nm. The effect and color changes of CV dye degradation are shown in Fig. 7. To determine the degradation, efficiency was observed using UV-Vis spectroscopy analysis which was shown in Fig. 8a and confirmed the surface plasmon resonance (SPR) band of CV at 580 nm. The efficiency of a nano catalyst was proved as 94% within 70 min which has been proved as a UV band at 580 nm. The CV rate of degradation is said to be 0.02848/min (Fig. 8b) which has been supported by first order kinetics. Finally, we have concluded that it is a time-dependent reaction (Fig. 8c and d).

**Fig. 9:**
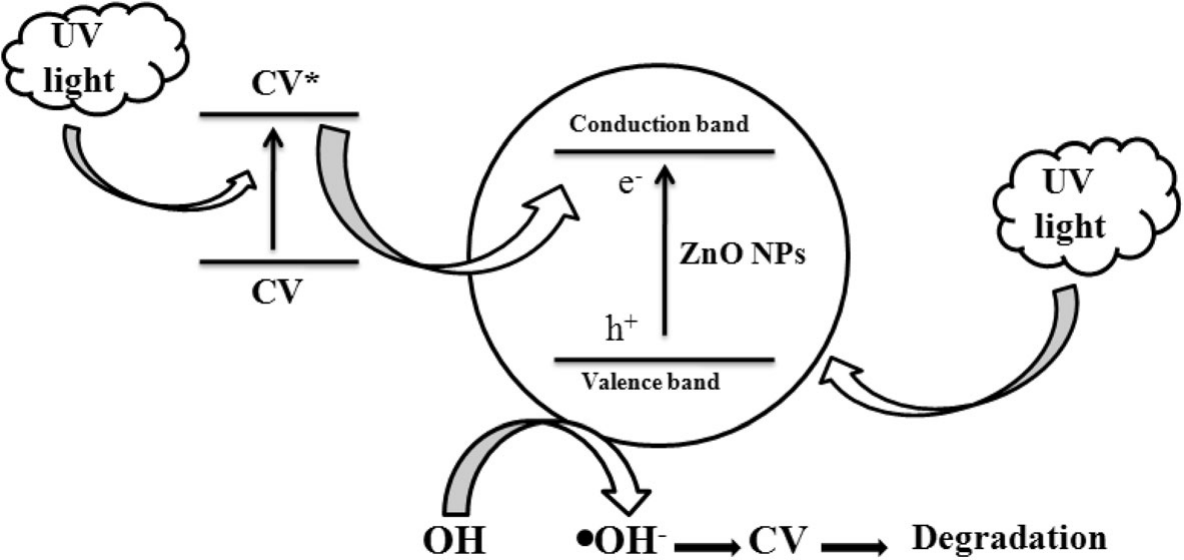
*Schematic diagram* of dye degradation mechanism of ZnO NPs

Figure 9 was proposed as a possible mechanism of photocatalytic activity of ZnO NPs against CV. The UV light absorption was extended by SPR band of ZnO NPs. Further, ZnO absorbs more energy photons than its band gap; due to this reason electrons are promoted to the CB from its VB. This leads to creating an equal number of holes in the VB [27]. Accordingly, ZnO NPs act as good photogenerated electrons to avoid the holes recombination with energy. This process is known as direct electron transfer and depends on the band structure of noble metal and metal oxide NPs. Furthermore, ZnO NPs participated in the degradation of CV dye using the UV light irradiation. The CV transfers the electrons into the ZnO CB [27]. This process leads to the reaction between photogenerated electrons and dissolved O_2_ and the formation of superoxide anion radicals [28–30].

3 $$ \mathrm{Z}\mathrm{n}\mathrm{O} + \mathrm{U}\mathrm{V}\ \mathrm{light}\ \left(365\ \mathrm{n}\mathrm{m}\right)\to \mathrm{Z}\mathrm{n}\mathrm{O}\ \left[{\mathrm{e}}^{-}\left(\mathrm{C}\mathrm{B}\right) + {h}^{+}\left(\mathrm{V}\mathrm{B}\right)\right] $$

4 $$ \mathrm{Z}\mathrm{n}\mathrm{O}\ \left[{h}^{+}\left(\mathrm{V}\mathrm{B}\right)\right] + {\mathrm{H}}_2\mathrm{O}\to \mathrm{Z}\mathrm{n}\mathrm{O} + {\mathrm{H}}^{+} + {\mathrm{OH}}^{\bullet } $$

5 $$ \mathrm{Z}\mathrm{n}\mathrm{O}\ \left[{e}^{-}\left(\mathrm{C}\mathrm{B}\right)\right] + {\mathrm{O}}_2\to {{\mathrm{ZnO} + \mathrm{O}}_2}^{-} $$

6 $$ {{\mathrm{O}}_2}^{-} + {\mathrm{H}}^{+}\to {\mathrm{H}\mathrm{O}}_2 $$

7 $$ {\mathrm{H}\mathrm{O}}_2 + {\mathrm{H}\mathrm{O}}_2\to {\mathrm{H}}_2{\mathrm{O}}_2 + {\mathrm{O}}_2 $$

8 $$ {\mathrm{H}}_2{\mathrm{O}}_2{{ + \mathrm{O}}_2}^{-}\to {\mathrm{O}\mathrm{H}}^{\bullet }+{\mathrm{O}\mathrm{H}}^{\mathrm{n}} + {\mathrm{O}}_2 $$

9 $$ \mathrm{Dye} + {\mathrm{OH}}^{\bullet}\to \mathrm{Degradation}\ \mathrm{product} $$

### ZnO NPs: Antifungal Activity

**Fig. 10:**
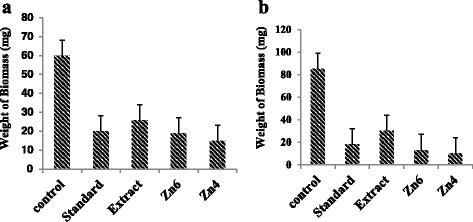
Antifungal activity on (**a**) *A. saloni* and (**b**) *S. rolfii* strains

It resulted in excellent in vitro antifungal activity on the two plant pathogens such as of the *A. saloni* and *S. rolfii*. The results were recorded for ZnO NPs at 400 °C, ZnO NPs at 600 °C, and methanolic extract of MFPE. The ZnO NPs at 400 °C showed efficient antifungal activity compared with ZnO NPs at 600 °C and methanol extract in both fungal strains. Figure 10 shows the antifungal activity of ZnO NPs at 400 °C, 600 °C, and methanol extract of MFPE on *A. saloni* and *S. rolfii* strains.

### In Vitro Hemolysis Activity of ZnO NPs

**Fig. 11:**
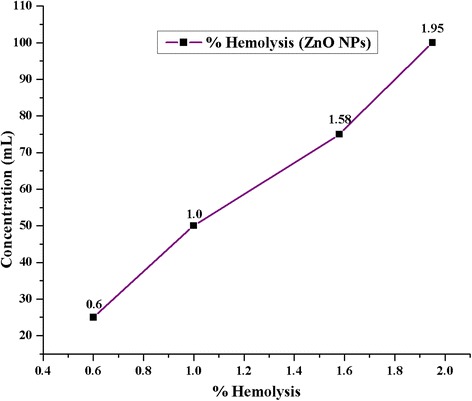
Hemolysis percentage of ZnO NPs

The activity to determine the hemolytic property of ZnO NPs at 400 °C showed the different percentage of ranges such as 1.95, 1.58, 1.00, and 0.6% for the different concentrations of 100, 75, 50, and 20 μL, respectively (Fig. 11). The results stated that ZnO NPs showed lower hemolytic activity compared with positive control. The mechanism of the hemolytic activity of the NPs depends on the increasing permeability to complete lysis of the cell. The cell lysis caused free radical formation and cell death [23] and resulted in harmless RBC cell count.

### ZnO NPs: Antibacterial Activity

**Fig. 12:**
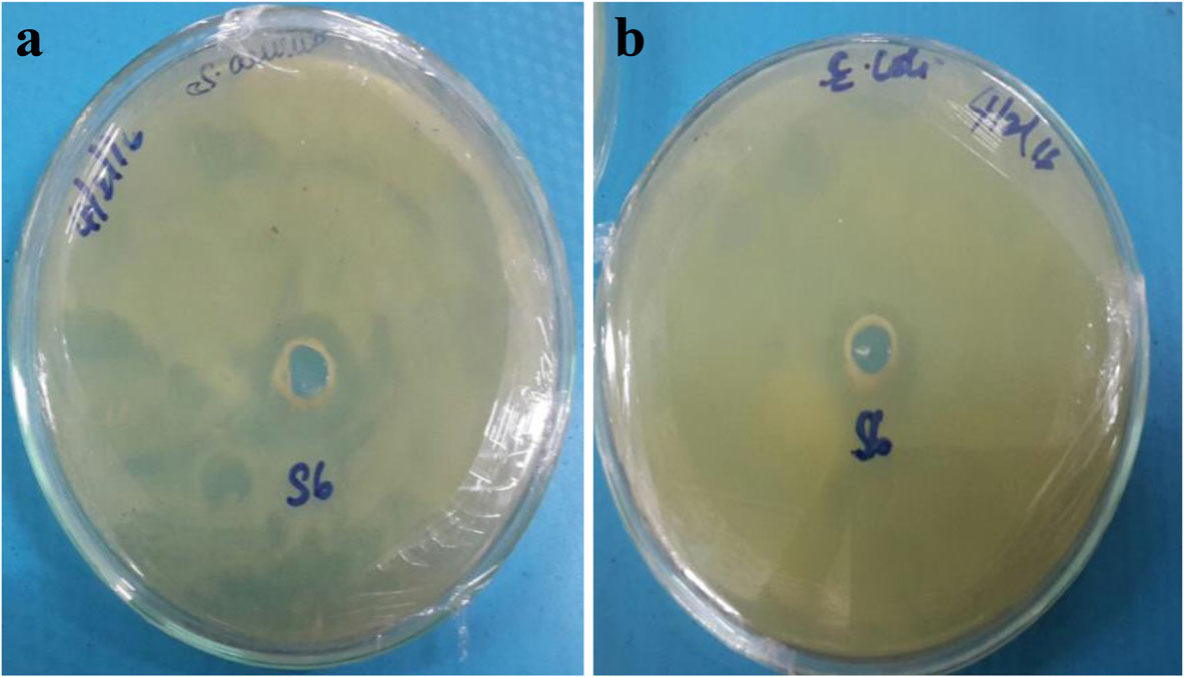
Antibacterial activity of ZnO NPs against bacterial strains (**a**) *S. aureus* and (**b**) *E. coli*

With the method zone of inhibition, antibacterial assays were calculated against *S. aureus* and *E. coli* bacterial strains (Fig. 12). The ZnO NPs, which are synthesized from MFPE, showed efficient antibacterial activity on both gram positive and gram negative bacterial strains [24]. The zone of inhibition of the ZnO NPs on *S. aureus* and *E. coli* is 6 mm.

The ZnO NPs have a good capacity to disrupt the bacterial cell membrane by ROS, e.g. super oxides and hydroxyl radical production. The surface of the ZnO NPs was also occupied with positive zeta potential; due to this, the particles can actively participate in the damage of bacterial cell membrane. This may show the impact decreases the cytoplasmic content on bacterial cells and leads to cell death [31]. According to this mechanism action, the synthesized ZnO NPs damaged the bacterial cell membrane and extrusion of cytoplasm was followed by cell death.

## Conclusions

In this research paper, we have synthesized the ZnO NPs from MFPE and determined the photocatalytic efficiency of ZnO NPs. The analytical data also resulted in an average size of 40–45 nm using XRD analysis. TEM analysis determined the morphology of ZnO NPs as spherical and hexagonal shapes. The photocatalytic activity increased due to its smaller particle size and ZnO NPS showed excellent photocatalytic activity at 365 nm. ZnO NPs at 400 °C showed good antifungal activity against both *A. saloni* and *S. rolfii* strains. Further, NPs were tested the effect of erythrocyte count and antibacterial activity on *S. aureus* and *E. coli*. The results stated that ZnO NPs synthesized from MFPE were non-toxic on RBCs and good antibacterial agents.

## Abbreviations

APC: Absorbance positive control
JCPDS: Joint committee powder diffraction standards
PBS: Phosphate buffered saline
TEM: Transmission electron microscope
ZnO NPs: Zinc oxide nanoparticles
